# Genetic variant in IL-32 is associated with the *ex vivo* cytokine production of anti-TNF treated PBMCs from rheumatoid arthritis patients

**DOI:** 10.1038/s41598-018-32485-0

**Published:** 2018-09-19

**Authors:** Michelle S. M. A. Damen, Kiki Schraa, Lieke Tweehuysen, Alfons A. den Broeder, Mihai G. Netea, Calin D. Popa, Leo A. B. Joosten

**Affiliations:** 1 Department of Internal Medicine, Radboud Centre for Infectious Diseases (RCI) and Radboud University Nijmegen Medical Centre, Nijmegen, The Netherlands; 2 Department of Rheumatology, Sint Maartenskliniek, Nijmegen, The Netherlands; 3 Human Genomics Laboratory, Craiova University of Medicine and Pharmacy, Craiova, Romania; 4 Department of Rheumatology, Radboud University Nijmegen Medical Centre, Nijmegen, The Netherlands; 5 Division of Immunobiology, Cincinnati Childrens Hospital Medical Center, Cincinnati Ohio, United States of America

## Abstract

About 60% of RA patients don’t achieve good response with biological disease-modifying anti-rheumatic drugs bDMARD treatment (including TNF inhibitors, TNFi’s). Previously, a link between TNFα and interleukin (IL)-32 was reported in RA. However, the exact mechanism linking IL-32 to response to treatment as not been studied yet. Therefore, we explored the influence of a promoter single nucleotide polymorphism (SNP) rs4786370 in IL-32 on clinical responsiveness to TNFi’s in RA patients, potentially serving as new biomarker in RA. Expression of pro-inflammatory cytokines by peripheral mononuclear cells (PBMCs) from RA patients and healthy individuals were studied. Moreover, “*ex vivo response*” and clinical response to anti-TNFα therapy (etanercept, adalimumab) were measured and stratified for the IL-32 SNP. Higher IL-32 protein production was observed in RA patients. Additionally, patients bearing the CC genotype showed higher IL-32 protein and cytokine expression. DAS28 was independent of the promoter SNP, however, the “*ex vivo”* cytokine response was not. IL-32 mRNA and protein production was higher in RA patients, with a trend towards higher concentrations in patients bearing the CC genotype. Furthermore, genotype dependent IL-1 beta production might predict clinical response to etanercept/adalimumab. This indicates that IL-32 could play a role in predicting response to treatment in RA.

## Introduction

Rheumatoid arthritis (RA) is a common autoimmune and chronic inflammatory disease affecting about 1% of the population^1,2^. RA is characterized by persistent joint inflammation, progressive disability and ongoing systemic inflammation, which can lead to joint deformity and low quality of life. Moreover, these characteristics are also able to increase the risk for atherosclerosis and cardiovascular disease (CVD), which is the main cause of death in these patients^3–5^. The clinical course of RA varies tremendously between patients from spontaneous remission, to mild joint symptoms to severe bone destruction. Early and aggressive treatment has been shown to improve the outcome. The introduction of biological disease-modifying anti-rheumatic drugs (bDMARDs) in combination with a “treat-to-target” treatment strategy significantly improved the disease outcomes in RA. These bDMARDs however still only achieve good response in about 40% of RA patients. The variation in clinical response to bDMARDs could be explained by variations in drug concentration and pharmacokinetics, which in turn are influenced by age, gender and renal or liver function^6^. Alternatively, the genetic background may also play a role and the interplay with the other factors could conduct towards specific profiles and increase the chance of achieving a good clinical response, suggesting a niche personalized medicine.

The pathogenesis of RA still remains partly unknown but results in a chronic inflammatory state. The initial phase involves the activation of T and B cells and the induction of pro-inflammatory cytokines such as IL-6, IL-1β and TNFα^7–9^. TNFα is clearly of high importance in the pathogenesis of RA, which is shown by the fact that TNFi’s can effectively reduce the chronic inflammation in RA^10,11^. Moreover, TNFα is also capable of inducing other pro-inflammatory mediators, such as chemokines and cytokines IL-6, IL-1β and IL-32, all found to be important in RA^11,12^. Studies of the last decade have shown that the cytokine interleukin-32 (IL-32) by itself is a strong inducer of TNFα and the expression levels of IL-32 in synovial biopsies correlated with inflammation severity in RA^13,14^. Moreover, overexpression of IL-32γ in human synovial fibroblasts followed by stimulation of TLR2/NOD2, showed a potent induction of TNFα mRNA^15^. In contrast, when IL-32 was- suppressed, TNFα production was decreased in human monocytes, all showing the important pro-inflammatory properties of IL-32 and its close relation with TNFα^15,16^. Despite knowing the interaction between these two cytokines and the importance in RA, research on the specific role of IL-32 in RA remains scarce. Our group recently showed a role for a promoter single-nucleotide polymorphism (SNP) in IL-32 that seemed to be associated with cytokine production, IL-32 isoform expression and high-density cholesterol (HDLc) levels in RA patients^17^. The present study therefore aims to investigate the possible predictive implications of this SNP in the IL-32 gene on the severity of the disease and the clinical response to TNFI’s (including adalimumab or etanercept) in RA patients.

## Results

### IL-32 isoform mRNA expression in RA patients versus healthy subjects

**Figure 1:**
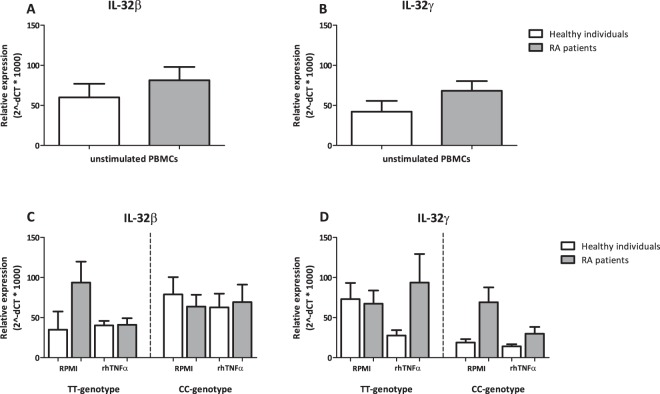
mRNA expression of IL-32 isoforms IL-32β, IL-32γ, in RA patients and healthy individuals in (un)stimulated PBMCs also separated on IL-32 promoter SNP genotypes. (**A**) mRNA expression of IL-32β in PBMCs from healthy individuals versus RA patients in unstimulated PBMCs (n = 7 healthy individuals; n = 22 RA patients). (**B**) mRNA expression of IL-32γ in PBMCs from RA patients and healthy individuals in unstimulated PBMCs (n = 7 healthy individuals; n = 22 RA patients). (**C**) mRNA expression of IL-32β in unstimulated PBMCs or PBMCs stimulated with rhTNFα from RA patients versus healthy individuals with the CC- versus TT-genotype for the IL-32 promoter SNP (n = 13 TT; n = 9 CC RA patients). (**D**) mRNA expression of IL-32γ in unstimulated PBMCs or PBMCs stimulated with rhTNFα from RA patients versus healthy individuals with the CC- versus TT-genotype for the IL-32 promoter SNP (n = 13 TT; n = 9 CC RA patients).

PBMCs of RA patients (n = 22) and those of healthy individuals (n = 7) were isolated and IL-32β and IL-32γ mRNA expression were determined. First, IL-32β and IL-32γ isoforms expression was determined in unstimulated PBMCs, independent of the IL-32 promoter SNP genotype (Fig. 1A,B). Although no significant differences were observed, there is a clear trend towards more IL-32β and IL-32γ mRNA expression in the RA patients compared to the healthy individuals (Fig. 1A,B). Thereafter we examined whether the IL-32 promoter SNP genotype (T/C) would influence this latter result (Fig. 1C,D). Unstimulated PBMCs of RA patients with the TT-genotype showed a tendency towards higher IL-32β mRNA expression compared to healthy individuals (Fig. 1C). RA patients bearing the CC-genotype did not show differences on IL-32β mRNA expression neither in unstimulated nor in the case PBMCs have been stimulated with recombinant human TNFα (rhTNFα) (Fig. 1C). Exploring specifically IL-32γ, a trend towards higher IL-32γ mRNA expression in unstimulated PBMCs of patients bearing the CC genotype could be observed. When stimulated with rhTNFα, RA patients bearing the TT-genotype seemed to express more IL-32γ compared to the unstimulated condition and the expression of IL-32γ in the group bearing the CC-genotype. However, none of these observations were statistical significant (Fig. 1D).

### IL-32 protein expression is highest in RA patients bearing the CC-genotype

**Figure 2:**
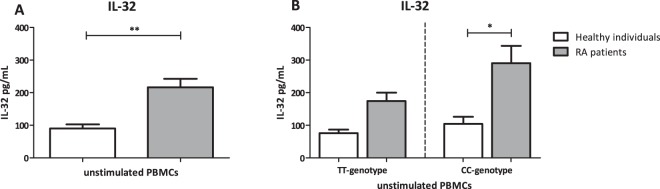
Protein expression of total IL-32 in unstimulated PBMCs from healthy individuals and RA patients also stratified for *IL32* promoter SNP. (**A**) A statistical significant difference was found in IL-32 protein expression between these groups (p = 0,0044) using the Mann-Whitney test in Graphpad Prism v5.03 (n = 8 TT healthy individuals and n = 25 TT RA patients versus n = 8 CC healthy individuals and n = 14 CC RA patients). (**B**) IL-32 protein concentrations were stratified for the IL-32 promoter SNP genotypes TT and CC. A significantly higher concentration of IL-32 protein was found in the RA patients carrying the CC-genotype compared to healthy individuals with the same genotype (p = 0.0105).

Even though there were no significant differences observed in IL-32β and IL-32γ isoforms mRNA expression between RA patients and healthy individuals, independent of IL-32 promoter SNP, the endogenous IL-32 protein expression was investigated. IL-32 protein concentration was determined in the two groups as a whole as well as stratified for IL-32 promoter SNP (Fig. 2A,B). Figure 2A shows a significantly higher IL-32 protein expression in RA patients compared to healthy individuals in unstimulated PBMCs. Of note, the production of IL-32 protein was significantly higher especially in the RA patients with the CC-genotype (Fig. 2B), whereas in the group bearing the TT-genotype it did not reach statistical significance.

### A pro-inflammatory status of RA PBMCs bearing the CC-genotype

**Figure 3:**
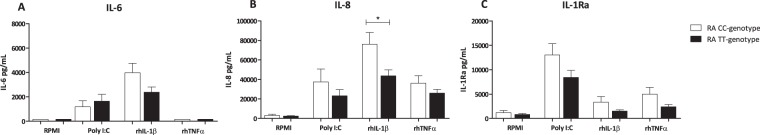
Cytokine measurements in PBMCs from RA patients and stratified for the IL-32 promoter SNP after stimulation with either Poly I:C and rhIL-1β or rhTNFα. (**A**) IL-6 protein concentration in PBMCs of RA patients with the TT- versus CC-genotype for the promoter SNP, at basal level and after stimulation. (**B**) IL-8 protein concentration in PBMCs of RA patients with the TT- versus the CC-genotype for the IL-32 promoter SNP, at basal level and after stimulation. Significant differences were observed after rhIL-1β stimulation between the CC- versus TT-genotype (p = 0.0335). (**C**) IL-1Ra protein concentration in PBMCs of RA patients with the TT- versus CC-genotype for the IL-32 promoter SNP at basal level as well as after stimulation. Statistical significance was shown by * when p < 0.05, GraphpadPrism V5.03. (n = 27 TT RA patients and n = 15 CC RA patients).

After observing differences in IL-32 mRNA isoforms expression and IL-32 protein expression between RA patients and healthy individuals, we examined the potential role of the CC-genotype in RA patients. We were interested to determine whether the genotypes in IL-32 promoter SNP would be associated with a different inflammatory response. In order to explore this, we investigated the capacity of *ex-vivo* cytokine production by PBMCs after exposure with various pro-inflammatory stimuli. Figure 3A,B clearly showed that PBMCs isolated from RA patients bearing the CC-genotype produce more IL-6 and IL-8, compared to TT genotype. Depending on the stimuli, the enhanced production of IL-8 reached statistical significance after PBMCs have been stimulated with rhIL-1β (Fig. 3B). In line with these results, PBMCs bearing the CC genotype produce more IL-1Ra, although this was not statistical significant.

### DAS28-CRP is not affected by IL-32 promoter SNP genotypes

**Figure 4:**
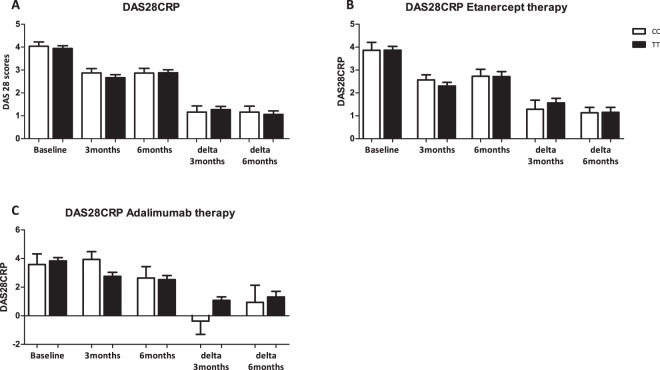
Disease activity scores (DAS) 28 in RA patients stratified on IL-32 promoter SNP and therapy used (etanercept or adalimumab). (**A**) DAS 28CRP separated on IL-32 promoter SNP genotype and independent of therapy (n = 37 CC vs 85 TT). (**B**) DAS28CRP separated on IL-32 promoter SNP genotype and Etanercept therapy (n = 13 CC vs 32 TT). (**C**) DAS28CRP separated on IL-32 promoter SNP genotype and Adalimumab therapy (n = 4 CC vs 13 TT).

After observing that RA patients, particularly those bearing the CC-genotype of the IL-32 SNP promoter, produce more pro-inflammatory cytokines, we thought to investigate whether this could have an impact on disease activity of these RA patients. To quantify disease activity, we used well-established DAS28-CRP score. DAS28-CRP scores were measured at baseline and after 3 and 6 months of follow-up. As shown in Fig. 4A, no differences were observed in DAS28-CRP scores between the two genotypes for the IL-32 promoter SNP at baseline. In the subgroups of patients who started treatment with respectively adalimumab or etanercept. IL-32 genotype did not influence the DAS28-CRP at three as well as six months afterwards (Fig. 4B,C).

### IL-32 genotype could help to predict the response on etanercept and adalimumab in RA

**Figure 5:**
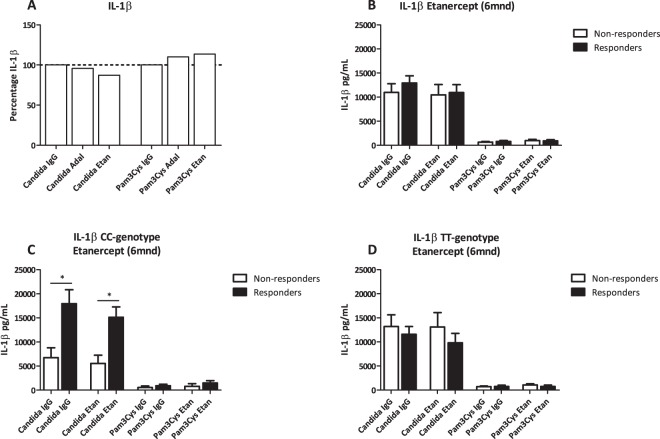
Percentage of IL-1β production corrected for baseline IL-1β production induced by Candida IgG stimulation of PBMCs from RA patients in the presence of anti-TNFα treatment (etanercept). (**A**) IL-1β cytokine production after *ex vivo* stimulation of PBMCs from RA patients with additional etanercept or adalimumab for 24 h independent of clinical treatment (n = 121). (**B**) IL-1β cytokine production after *ex vivo* stimulation of PBMCs from RA patients with additional etanercept, stratified for RA patients that clinically responded or not to etanercept (n = 26 non-responders; n = 19 responders). (**C**) IL-1β cytokine production after *ex vivo* stimulation of PBMCs from RA patients with additional etanercept, stratified for clinical responders to etanercept treatment and IL-32 promoter SNP (n = 4 CC; n = 15 TT RA patients). Statistical significant differences were observed after Candida IgG (p = 0.0336) stimulation and Candida Etan (p = 0.0196). (**D**) IL-1β cytokine production after *ex vivo* stimulation of PBMCs from RA patients with additional etanercept, stratified for clinical non-responders to etanercept and IL-32 promoter SNP genotype (n = 9 CC and n = 17 TT RA patients). Statistical significance was shown by * when p < 0.05, GraphpadPrism V5.03.

In order to provide a possible explanation for the fact that the IL-32 promoter SNP did not influence the response of RA patients to therapy with adalimumab or etanercept in the first six months, we further thought to assess whether the genotype could affect the *ex vivo* response of PBMCs to various stimuli in the presence of etanercept or adalimumab. In addition, we also wanted to address the question whether patients who eventually show a good clinical response to these bDMARDs have a different inflammatory response at baseline dependent on the IL-32 genotype. More specifically, if RA patients with either the CC or TT genotype would respond better to the specific bDMARD by showing a different cytokine response (Fig. 5A–D and Supplementary Fig. S1 and Tables T1, T2). IL-1β production by PBMCs tends to decrease after *Candida albicans* stimulation in the presence of either adalimumab or etanercept (Fig. 5A). When the IL-1β cytokine production was stratified for clinical responders versus non-responders to either etanercept or adalimumab (Figs 5B and Supplementary S1), higher IL-1β production in the clinical responders was noted, though not reaching statistical significance. We further divided these groups according to their genotype for the IL-32 promoter SNP. Figure 5C,D and Supplementary Fig. S1 indicate that for the clinical responders to either etanercept or adalimumab, RA patients bearing the CC genotype seem to produce more IL-1β after *ex* vivo stimulation of PBMCs, even reaching a statistical significant difference in the case of etanercept treatment. In contrast, no such effect was observed for RA patients bearing the TT genotype. To try and explain this effect in more detail we calculated the percentage of *ex vivo* responders to etanercept and adalimumab. This data however does not show to be able to predict a possible effect of the IL-32 promoter SNP on the clinical responsiveness of RA patients to anti-TNFα therapy (Supplementary Tables T1, T2).

## Discussion

In this study, we showed that mRNA expression levels of IL-32 isoforms IL-32β and IL-32γ tended to be higher in PBMCs isolated from RA patients as compared to healthy individuals. Additionally, intracellular IL-32 protein production was higher in RA patients, especially in those bearing the CC-genotype. These patients also tended to produce more pro-inflammatory cytokines *in-vitro*. However, this fact did not translate into a higher disease activity, as assessed by DAS28-CRP scores. Finally, *ex-vivo* PBMCs stimulations in the presence of the intended medication could potentially identify the patients who would become responders according to clinical criteria. The determination of IL-32 promoter SNP in this context proved to have crucial importance.

IL-32 mRNA isoforms have been studied so far in many different cell types and diseases including chronic obstructive pulmonary disease (COPD), various types of cancer and RA^14,18–20^. Nevertheless, the exact expression pattern of the isoforms in specific cells or tissues and the function of each isoform still need to be elucidated^20^. It is known that the structure of the two isoforms IL-32β and IL-32γ is quite similar^21^. Both isoforms seem to be involved in pro-inflammatory processes, including the induction of other pro-inflammatory cytokines and chemokines like IL-8, IL-6, IL-1β and TNFα. In our study, we found a higher IL-32 protein production from PBMCs of RA patients together with a slightly higher IL-32 mRNA expression in the same patients. This is in line with previous data pointing to a higher IL-32 mRNA expression in fibroblast-like synoviocytes (FLS) of RA patients and also in the PBMCs^21,22^. Interestingly, here we show that only in patients bearing the CC-genotype, the increase in plasma IL-32 concentrations reaches statistical significance. This could also be due to the low number of patients included for this analysis. Alternatively, this might suggest that the studied SNP in IL-32 promoter has functional consequences. Finally, a difference between isoforms expression has been observed within the patients, which varied from the expression pattern seen in healthy individuals.

In a previous study, we indicated that the promoter SNP is associated with different levels of high-density lipoprotein cholesterol (HDLc) concentrations in RA patients, namely higher HDLc concentrations^17^. These results already suggested that this SNP might be functional. In the present report, we extended our observations at the level of cytokine production and immune responses. PBMCs from RA patients showed a difference in cytokine production when the patients were separated according to their genotype. Our study is the first to show that and therefore confirms the fact that the promoter SNP serves a functional effect.

Given the contribution of IL-32 and TNF to the inflammatory cascade in RA, we further investigated whether the SNP in IL-32 promoter region impacts disease activity in these patients. Absolute levels of disease activity are set to define the disease state of the patients and over time can be used to define improvement or response to treatment^23^. Finding a marker that could predict the latter would therefore be useful, although current treat to target treatment strategies already have very good long term outcomes. Unfortunately, this study showed that the promoter SNP in IL-32 was not such a marker. Even though it has functional effects on cytokine production by PBMCs or on the HDLc concentration, no effect was seen on the level of disease activity, as measured by DAS28-CRP. These results are somehow in contrast with the study of Gui *et al*., which observed correlations between the IL-32 levels and disease activity^22^. The number of patients enrolled and the different design might explain this discrepancy. Alternatively, IL-32 might differently impact the various components of DAS28 score, for instance the number of tender joints or the VAS.

Single nucleotide polymorphisms in other cytokines such as TNFα, IL-1β and IL-6 have been studied in possible association to response to treatment in RA patients^24–27^. Since IL-32 is strongly linked to TNFα expression and can induce IL-6 and IL-1β, it was worth investigating whether this SNP also had an effect on clinical response to treatment. No differences were observed when looking at the clinical response independent of treatment or anti-TNFα (etanercept or adalimumab) treatment in particular, when separated on the promoter SNP for IL-32.

Besides the clinical response, the *ex vivo* response of PBMCs stimulated with additional etanercept or adalimumab and *Candida albicans* or Pam3Cys was studied by looking at IL-1β cytokine production after stimulation. A previous study by Popa C *et al*. could not detect differences in IL-1β cytokine production after anti-TNFα treatment^28^. Interestingly, our data however showed that PBMCs of RA patients bearing the CC genotype produced more IL-1β after *ex vivo* stimulation only within the group of RA patients that clinically responded to either etanercept or possibly adalimumab treatment. No such differences were observed either for the clinical non-responders or patients bearing the TT genotype. This was in line with data from another study by Kayakabe K *et al*. which showed that IL-1β could serve as a possible predictive measurement for response to anti-TNFα treatments^29^.

These results suggest that even though the IL-32 promoter SNP does not have an effect on DAS28 scores, there might be a role for the genetic polymorphism in the promoter region of IL-32 in the prediction of clinical response to anti-TNFα treatment in RA patients also taking into account the observed influence on the production of pro-inflammatory cytokines in these patients. Given the previously indicated relation between the promoter SNP and HDLc levels in RA patients and the new findings of the functional effect of the promoter SNP on cytokine production and response to treatment, these data show a possible additional role of IL-32 and its promoter SNP in RA. Our results therefore lead to an area of research in which the effects of IL-32 and the promoter SNP in RA have to be studied relevant to response to treatment and cardiovascular diseases within these patients. In line with this, we are currently performing studies to further elucidate the mechanisms behind the role of IL-32 in cholesterol metabolism regulation.

Some limitations could be envisaged in our study. One of the most important one might be related to the low number of patients per genotype (TT vs CC) for the IL-32 promoter SNP, especially in the analysis of the data concerning only patients who received etanercept or adalimumab. This is caused by the multiple stratifications e.g. genotypes, clinical responses; medications/treatments that were performed. This could interfere with statistical analysis and power in case a small difference in response to treatment would have been observed and sample size is small. In addition, healthy controls were not matched regarding age or gender to the patients group. Moreover, differences in cytokine production of TNFα were not measured directly and because of multiple freeze-thaw cycles, this was no longer possible. Therefore, this study lacks a very important cytokine measurement within this group of patients.

In conclusion, the present study shows that IL-32 mRNA and protein production was higher in RA patients compared to healthy individuals. Moreover, this study is the first to show a slightly higher IL-32 expression in patients bearing the CC-genotype for the IL-32 promoter SNP (rs4786370). Additionally, the promoter SNP tended to be associated with an increased expression of pro-inflammatory cytokines IL-6 and IL-8 produced by PBMCs of RA patients. These findings add to the previously described functional effect of the IL-32 promoter SNP on HDLc concentrations within RA patients. Nevertheless, we were unable to show an association between the promoter SNP and disease activity and clinical response to adalimumab and etanercept. However, most interestingly, we were able to show a link between the promoter SNP and the *ex vivo* induced cytokine production of IL-1β in RA patients that clinically responded to etanercept (or adalimumab). Therefore, we suggest that the exploration of the role of IL-32 in RA should be continued in future research, focussing more specifically on treatment response, inflammation induced cardiovascular disease burden and cholesterol metabolism abnormalities.

## Material and Methods

### Patient cohort

Blood samples were obtained at baseline from patients included in the prospective longitudinal prediction cohort study BIO-TOP [Biologic Individual Optimized Treatment Outcome Prediction] and isolated immune cells were used for various assays. RA patients >18 years, treated in the Sint Maartenskliniek (Nijmegen, the Netherlands) who were going to start with (or switch to) a biological disease-modifying anti-rheumatic drug (bDMARD) were included in this study. The local ethical committee (CMO region Arnhem-Nijmegen, NL47946.091.14) was responsible for approval of the BIO-TOP study and a detailed description is available in the Dutch trial register (NTR4647 clinical trial, 17-jun-2014, NTR). Additionally, blood samples of healthy individuals were used and obtained on the same day of that of a patient. Written informed consent was received from all donors. Experiments with human blood were performed in accordance with the Declaration of Helsinki.

### DNA isolation and taqman genotyping

Whole blood obtained from 329 RA patients in the BIOTOP study was used to perform genomic DNA extraction. Genomic DNA was isolated from whole blood using the Qiagen (Valencia, CA, USA) isolation kit and following the standard protocol. The samples were quantified and evaluated for purity (260/280-nm ratio) with a NanoDrop ND-1000 spectrophotometer (Thermo Scientific). Using the Taqman SNP assay C_27972515_20 (Thermofisher, Foster City, CA, USA), each genetic variant in the *IL32* promoter (rs4786370) polymorphism was determined. The TaqMan qPCR assays were performed on the AB StepOnePlus polymerase chain reaction system (Applied Biosystems). Negative controls were included in the assay. No duplicates were used.

### Isolation and e*x-vivo* stimulation of peripheral blood mononuclear cells (PBMCs)

At baseline (before start bDMARD), venous blood was collected into three 10 mL EDTA tubes after informed consent. Within 24 hours, PBMCs were isolated by density gradient centrifugation using Ficoll-Paque PLUS (GE Healthcare, Zeist, The Netherlands) and collecting the buffy-coat enriched layer. Cells were washed twice in cold PBS and concentrations were adjusted to 5 × 10^6^ cells/ml in RPMI-1640, supplemented 2 mM l-glutamine, 1 mM pyruvate and 50 μg/ml gentamicin (GIBCO Invitrogen, Carlsbad, CA). Mononuclear cells (5 × 10^5^) in a 100-μl volume were added to round-bottom 96-well plates (Greiner, Nurnberg, Germany) and incubated with either 100 μl of culture medium (negative control), Poly I:C (50uL Invivogen), IL-1β (1 ng/mL R&D Systems), or recombinant human TNFα (rhTNFα 10 ng R&D Systems). After 24 hour incubation at 37 °C, the supernatants were stored at −20 °C until further use. Besides these experiments, additionally 5 × 10^5^ PBMCs were pre-incubated in round bottom 96-well plates for one hour at 37 °C with therapeutic *in vivo* concentrations of adalimumab or etanercept. Taking into account the different half-life times, dosing and treatment intervals, and therapeutic concentration ranges of the anti-TNFα, the same concentration of 5 µg/mL was added for both anti-TNFα bDMARDS (4–6). Human IgG was used as negative control. Thereafter, cells were stimulated with either Pam3Cys (a TLR2 agonist) or heat killed *Candida albicans* (ATCC MYA-3573 (UC 820)). After 24 hours, supernatants were stored at −20 °C until assayed.

### Cytokine measurements

Various cytokines were determined in supernatant after stimulation of PBMCs with recombinant human TNFα (10 ng/ml) (R&D Systems), recombinant human IL-1β (1 ng/mL R&D Systems) or Poly I:C (TLR3 agonist) (50 µg/ml) (Invivogen) for 24 hours, by commercially available ELISA kits according to manufacturer’s instructions. Concentrations of human IL-1β, IL-1Ra (R&D Systems, Inc., Minneapolis, MN, USA) and IL-6, IL-8, IL-10 (Sanquin Reagents, Amsterdam, The Netherlands) were measured. IL-32 production was measured in cell lysates (Triton X 100 0.5%) of PBMCs stimulated with various ligands, using the commercially available ELISA kit (R&D Systems, Inc, Minneapolis, MN, USA). In brief, Maxisorp plates (Nunc) were coated with AF3040 (R&D Systems) diluted in Phosphate Buffered Saline (PBS) at a concentration of 0.4 μg/ml and incubated overnight at room temperature. Plates were blocked with PBS containing 1% BSA (Sigma-Aldrich) for 1 hour at room temperature. The standard curve was prepared by diluting recombinant IL-32 ranging from 5000 pg/ml until 39.06 pg/ml in PBS containing 5% BSA. After a 2 hour incubation with the cell lysates, detection antibody was added (BAF3040, R&D Systems), 0.1 µg/ml in PBS with 5% BSA for 1 hour. Streptavidin (R&D Systems) was added for 30 minutes at room temperature after which substrate buffer was used to develop a color reaction that was measured by a plate reader.

### Clinical assessments

Disease activity was measured with the 28-joints disease activity score using C-reactive protein (DAS28-CRP) during outpatient clinical visits performed in usual care after 3 and 6 months (±1 month). Primary outcome was the DAS28-CRP based European League Against Rheumatism (EULAR) response criteria (good versus moderate/no response) at month 6.

### Statistical analysis

Normality was tested using the D’Agostino normality test. Continuous variables are presented as mean and standard deviation (SD). The differences IL-32 mRNA expression, IL-32 protein concentrations and cytokine concentrations were analyzed using the Mann-Whitney U-test. A *p-*value less than 0.05 was considered statistically significant (*p < 0.05 and **p < 0.01). Data was analyzed using GraphPad Prism v5.3.

## Electronic supplementary material

Supplementary figure S1
