# Supplementary material for: Genetic variant in IL-32 is associated with the ex vivo cytokine production of anti-TNF treated PBMCs from rheumatoid arthritis patients
Source: Sci Rep. 2018 Sep 19;8:14050. doi: 10.1038/s41598-018-32485-0 (PMC6145899; doi:10.1038/s41598-018-32485-0)
Supplement: Supplementary file 1 — Supplementary figure S1 [file 41598_2018_32485_MOESM1_ESM.pdf]

Supplementary figure/table legends

Genetic variant in IL-32 is associated with the ex vivo cytokine production of anti-TNF treated PBMCs from rheumatoid arthritis patients

Michelle S.M.A. Damen[1], Kiki Schraa[1], Lieke Tweehuysen[2], Alfons A. den Broeder[2], Mihai G. Netea[1,3], Calin D. Popa[2,4], Leo A.B. Joosten[1,*]

Supplementary Figure

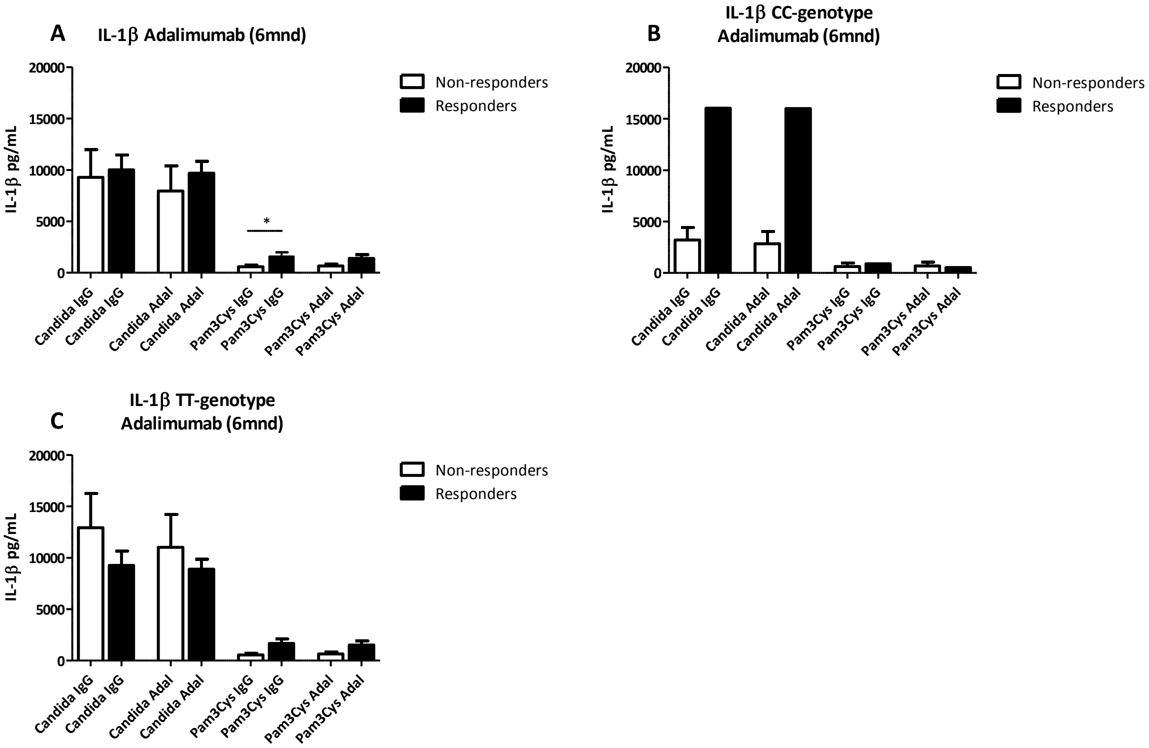

Figure S1. Production ofIL-1β induced by Candida IgG stimulation of PBMCs from RA patients in the presence of anti-TNFα treatment (adalimumab). A) IL-1β cytokine production after *ex vivo* stimulation of PBMCs from RA patients with additional adalimumab, stratified for RA patients that clinically responded or not to adalimumab (n=8 non-responders; n=9 responders). B) IL-1β cytokine production after *ex vivo* stimulation of PBMCs from RA patients with additional adalimumab, stratified for clinical responders to adalimumab treatment and IL-32 promoter SNP (n=1 CC; n=8 TT RA patients). C) IL-1β cytokine production after *ex vivo* stimulation of PBMCs from RA patients with additional adalimumab, stratified for clinical non-responders to adalimumab and IL-32 promoter SNP genotype (n=3 CC and n=5 TT RA patients).

Supplementary Table T1

Clinical Responders to Etanercept	CC	TT
	**Candida Etan**	**Candida Etan**
**IL-1β**	84,40	84,88
**Percentage red (non-responders)**	**25,00**	**26,67**
**Percentage green (responders)**	**75,00**	**73,33**
	**Pam3Cys Etan**	**Pam3Cys Etan**
**IL-1β**	163,57	97,26
**Percentage red (non-responders)**	**100**	**40,00**
**Percentage green (responders)**	**0,00**	**53,33**

TableT1. Overview of the percentage of IL-1β production corrected for *Candida* IgG or Pam3Cys IgG (as 100%) *ex vivo* stimulation in PBMCs of RA patients clinically responding to etanercept treatment and stratified for the IL-32 promoter SNP (n=4 CC versus n=15).

Supplementary Table T2

Clinical responders to Adalimumab	CC	TT
	Candida Adal	Candida Adal
**IL-1β**	99,74	95,95
Percentage red (non-responders)	0	62,50
Percentage green (responders)	100	37,50
	Pam3Cys Adal	Pam3Cys Adal
**IL-1β**	59,37	91,05
Percentage red (non-responders)	0	25,00
Percentage green (responders)	100	62,50

TableT2. Overview of the percentage of IL-1β production corrected for *Candida* IgG or Pam3Cys IgG (as 100%) *ex vivo* stimulation in PBMCs of RA patients clinically responding to adalimumab treatment and stratified for the IL-32 promoter SNP (n=1 CC versus n=8).
